# Tissue‐Engineered Tracheal Replacement in a Child: A 4‐Year Follow‐Up Study

**DOI:** 10.1111/ajt.13318

**Published:** 2015-06-02

**Authors:** N. J. Hamilton, M. Kanani, D. J. Roebuck, R. J. Hewitt, R. Cetto, E. J. Culme‐Seymour, E. Toll, A. J. Bates, A. P. Comerford, C. A. McLaren, C. R. Butler, C. Crowley, D. McIntyre, N. J. Sebire, S. M. Janes, C. O'Callaghan, C. Mason, P. De Coppi, M. W. Lowdell, M. J. Elliott, M. A. Birchall

**Affiliations:** ^1^ University College London Ear InstituteRoyal National Throat Nose and Ear HospitalLondonUK; ^2^ Department of Cardiothoracic SurgeryGreat Ormond Street HospitalLondonUK; ^3^ Department of RadiologyGreat Ormond Street HospitalLondonUK; ^4^ Department of OtorhinolaryngologyGreat Ormond Street HospitalLondonUK; ^5^ Imperial College London, Department of AeronauticsLondonUK; ^6^ London Regenerative Medicine NetworkLondonUK; ^7^ Lungs for Living Research CentreRayne InstituteLondonUK; ^8^ University College London Centre for Nanotechnology and Regenerative MedicineRoyal Free HospitalLondonUK; ^9^ Department of HistopathologyGreat Ormond Street HospitalLondonUK; ^10^ Department of Respiratory MedicineGreat Ormond Street HospitalLondonUK; ^11^ Department of SurgeryGreat Ormond Street HospitalLondonUK; ^12^ Department of HaematologyRoyal Free Hospital, University College London Paul O'Gorman Laboratory of Cellular TherapeuticsLondonUK

**Keywords:** Graft survival, growth and development, tissue/organ engineering

## Abstract

In 2010, a tissue‐engineered trachea was transplanted into a 10‐year‐old child using a decellularized deceased donor trachea repopulated with the recipient's respiratory epithelium and mesenchymal stromal cells. We report the child's clinical progress, tracheal epithelialization and costs over the 4 years. A chronology of events was derived from clinical notes and costs determined using reference costs per procedure. Serial tracheoscopy images, lung function tests and anti‐HLA blood samples were compared. Epithelial morphology and T cell, Ki67 and cleaved caspase 3 activity were examined. Computational fluid dynamic simulations determined flow, velocity and airway pressure drops. After the first year following transplantation, the number of interventions fell and the child is currently clinically well and continues in education. Endoscopy demonstrated a complete mucosal lining at 15 months, despite retention of a stent. Histocytology indicates a differentiated respiratory layer and no abnormal immune activity. Computational fluid dynamic analysis demonstrated increased velocity and pressure drops around a distal tracheal narrowing. Cross‐sectional area analysis showed restriction of growth within an area of in‐stent stenosis. This report demonstrates the long‐term viability of a decellularized tissue‐engineered trachea within a child. Further research is needed to develop bioengineered pediatric tracheal replacements with lower morbidity, better biomechanics and lower costs.

## Abstract 2

This case presents a 4‐year follow‐up of a tissue‐engineered pediatric tracheal transplant and reports on the longterm viability of this approach and areas for further research.

**Abbreviations**
GMP: good manufacturing practice
GOSH: Great Ormond Street Hospital
ICU: intensive care unit

## Introduction

The application of tissue engineering to restore a segment of airway in those with long‐segment tracheal stenosis resistant to treatment is now realistic. Such therapies remain highly controversial, however, due to uncertainty regarding long‐term benefit, quality of life and comparative cost. A 5‐year follow‐up of the first tissue‐engineered airway in an adult reported normal lung function and a good quality of life long‐term albeit with the need for serial insertions of airway stents 1, 2. Approaches based on synthetic scaffolds have also been reported, though only with short‐term follow‐up to date 3.

In 2012, we reported a 2‐year follow‐up of the first tissue‐engineered tracheal transplant in a child using a decellularized deceased donor trachea repopulated with the patient's own respiratory epithelial cells and mesenchymal stromal cells 4. While this demonstrated the validity of this approach in reconstructing an airway over a 2‐year period, a number of key questions remain unanswered. The trachea continues to grow up to adulthood and it is still not known whether a transplanted engineered airway has capacity for growth 5. The long‐term outcome is also unclear in terms of how the graft remodels over time, whether it continues to support regenerated epithelium and if it continues to be unchallenged by the immune system. An evaluation of the quality of life of the recipient, a chronology of events and estimation of cost is necessary to evaluate the potential for a wider application of this technology and its place within a healthcare system. We address these questions by reporting observations of this child's clinical progress and tracheal epithelialization 4 years after implantation.

## Materials and Methods

Clinical data were collated from the patient notes documenting his long‐term care at Great Ormond Street Hospital (GOSH). The Peds QL parent‐proxy assessment tool was used as a validated method of analyzing health‐related quality of life in children with chronic disease and associated learning disabilities 6.

Neck and chest computed tomography scans at 3 months (June 2010), 42 months (September 2013) and 49 months (April 2014) following transplantation were compared. 3D reconstructions of the tracheal lumen were performed using Mimics^®^ Innovation Suite (Materialise, Materialise Belgium – Technologielaan 15, Leuven, Belgium). Cross‐sectional area of the trachea was calculated following methods previously described 7. Tracheal airflow was calculated using a validated method through computational fluid dynamics 8, 9.

Endoscopy images of the transplanted trachea were obtained during microlaryngobronchoscopy performed at 15 days, 6, 15 and 42 months following transplantation using a 15‐mm zero degree Hopkins Rod and image capture (Karl Storz, Tuttlingen, Germany).

Biopsy samples were routinely processed following formalin fixation, followed by paraffin wax embedding and cutting of 4‐μm sections onto glass slides for hematoxylin and eosin (H&E) staining and immunostaining using an automated immunostainer (Leica Bond‐Max, Leica, Wetzlar, Germany). Samples of cilia were obtained during routine flexible bronchoscopy by brushing the trachea with a 2‐mm cytology brush (Olympus Endotherapy, Olympus, Skinjuku‐ku, Tokyo) 10. Samples were analyzed using a digital high‐speed video camera (MotionPro X4, IDT, CA) at a frame rate of 500 frames s–1 to allow assessment of ciliary beat frequency and pattern.

The costing for the clinical items were obtained using a combination of NHS reference costs (2010–2011 period) per procedure appropriate for a male child and typical costs for complex tracheal patients provided by GOSH. The laboratory costs were estimated from data gathered from the good manufacturing practice (GMP) laboratories where further grafts were constructed to treat two later patients (data not shown).

## Results

### Chronology of events

**Figure 1:**
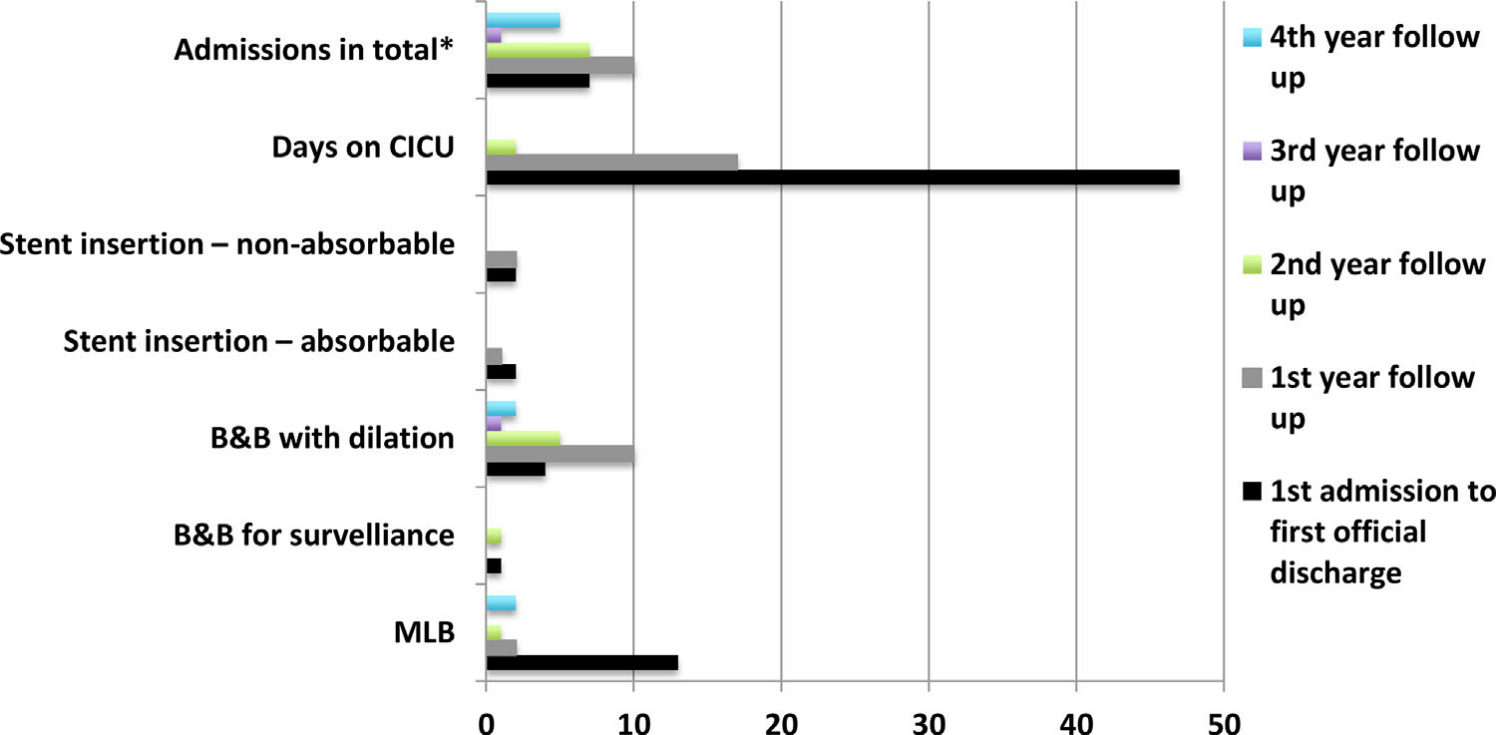
**Number of clinical events from transplantation to the fourth year of follow‐up.** The frequency of interventions fell significantly following the first year after transplantation. B&B, bronchoscopy and bronchogram; CICU, cardiac intensive care unit; MLB, microlaryngoscopy and bronchoscopy.

Following transplantation at GOSH in March 2010, the child returned to the intensive care unit (ICU; unit providing level 2 and 3 care) where he remained an inpatient for 8 days, before being discharged from critical care 11. He required 25 procedures postoperatively, mainly to clear secretions and granulation tissue (Figure 1). The graft itself was malacic in the initial period necessitating the insertion of two bioabsorbable tracheal stents. On four occasions during the initial period admission to the ICU was needed for respiratory support.

The child was discharged home in August 2010, but required a number of return visits to GOSH to address retained secretions, granulation and a malacic segment in the distal transplanted trachea, necessitating the insertion of further stents, one bioabsorbable and two self‐expanding nitinol. There were two further admissions to ICU during the first year follow up period and one ICU admission during the second year follow up period.

By 6 months, the airway was sufficiently stable to allow the child to return to school and the need for repeated interventions declined up to late 2013 when an infection and stenosis within the tracheal stents and un‐transplanted left main bronchus necessitated intervention. Following balloon dilatation of the trachea and left main bronchus, the child made a good recovery and has now returned to full‐time education.

A Peds QL 4 · 0 parent proxy quality of life assessment was completed in May 2014. Scores were 50, 60, 55 and 35 for physical, emotional, social and school subscales, respectively, with a total score of 50 out of 100. Since transplantation, the child has grown 15 cm in height to 168 cm and gained 21 kg in weight to 58 kg.

### Assessment of the transplanted trachea

**Figure 2:**
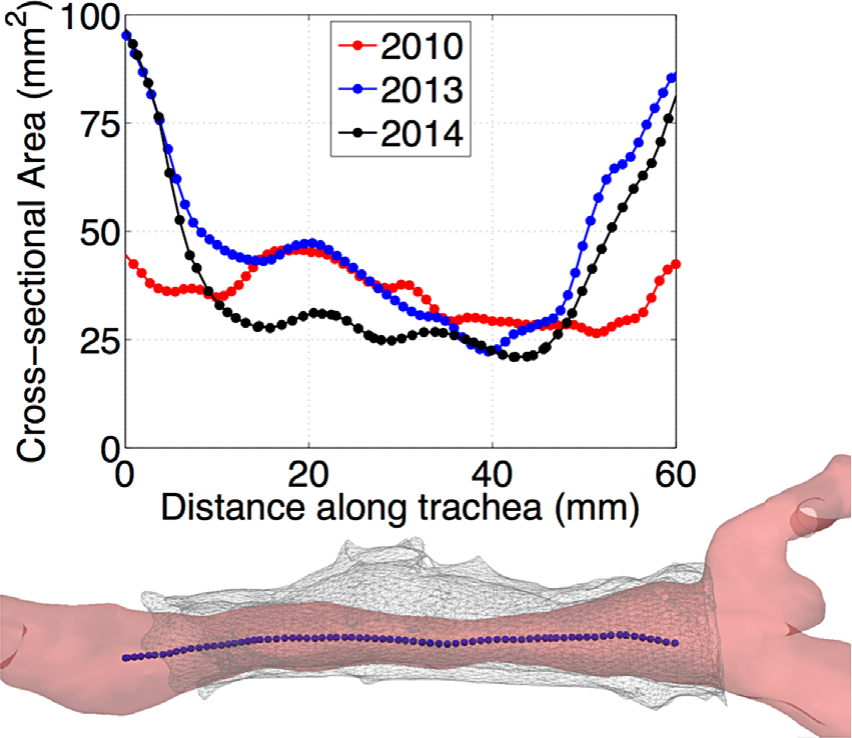
**Cross‐sectional variation in area along the length of the trachea as derived from each scan.** Both the more recent measurements demonstrate growth at either end of the transplanted section, whereas the 2010 measurements are more constant along the length. The minimum area has reduced by 20.5% between 2010 and 2014, while the area has more than doubled at the extremities. Below the area plot you can see a reconstruction of the 2013 geometry and stent with the centerline highlighted.

**Figure 3:**
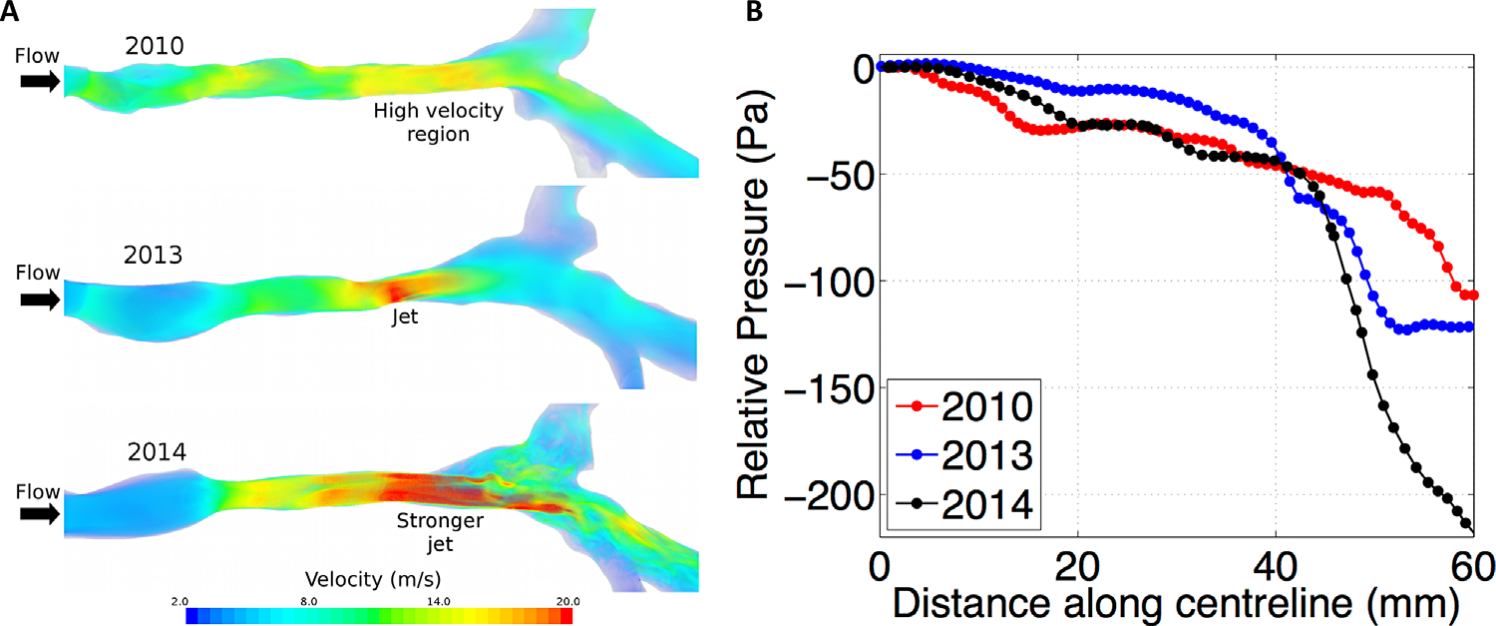
(A) Flow velocities. The velocity in the 2010 geometry is relatively uniform, similar to that of a healthy/normal individual in the upper region, while somewhat accelerated in the lower part. By 2013, a constriction is apparent which leads to the formation of a jet in that area. A longer constriction is seen in 2014 geometry which causes higher velocities in the upper region and a stronger jet below necessitating intervention with balloon dilatation. (B) Relative pressure. Here, we see relative pressure plotted along the distance of the centerline. 2013 shows an abrupt drop in pressure at the location of the jet, which then plateaus, to a pressure drop slightly higher than that in 2010. The long constriction and strong jet in 2014 results in double the pressure drop when compared to the 2010 geometry.

Figure 2 shows the cross‐sectional area comparison derived from the 2010, 2013 and 2014 CT scans. Comparison of the 2010 tracheal geometry to a normal trachea demonstrates the formation of high velocity region in its distal segment (Figure 3A) 12. In 2013, the velocity in this region increased further and formed a jet 40 mm below the glottis. By 2014, the jet formed more proximally (circa 17 mm subglottically). The effect of these changes in flow pattern is seen in Figure 3B. Lung Function Tests in October 2013 and April 2014 showed flattening of both inspiratory and expiratory parts of the inspiratory/expiratory flow volume curves consistent with a fixed obstruction of the trachea.

**Figure 4:**
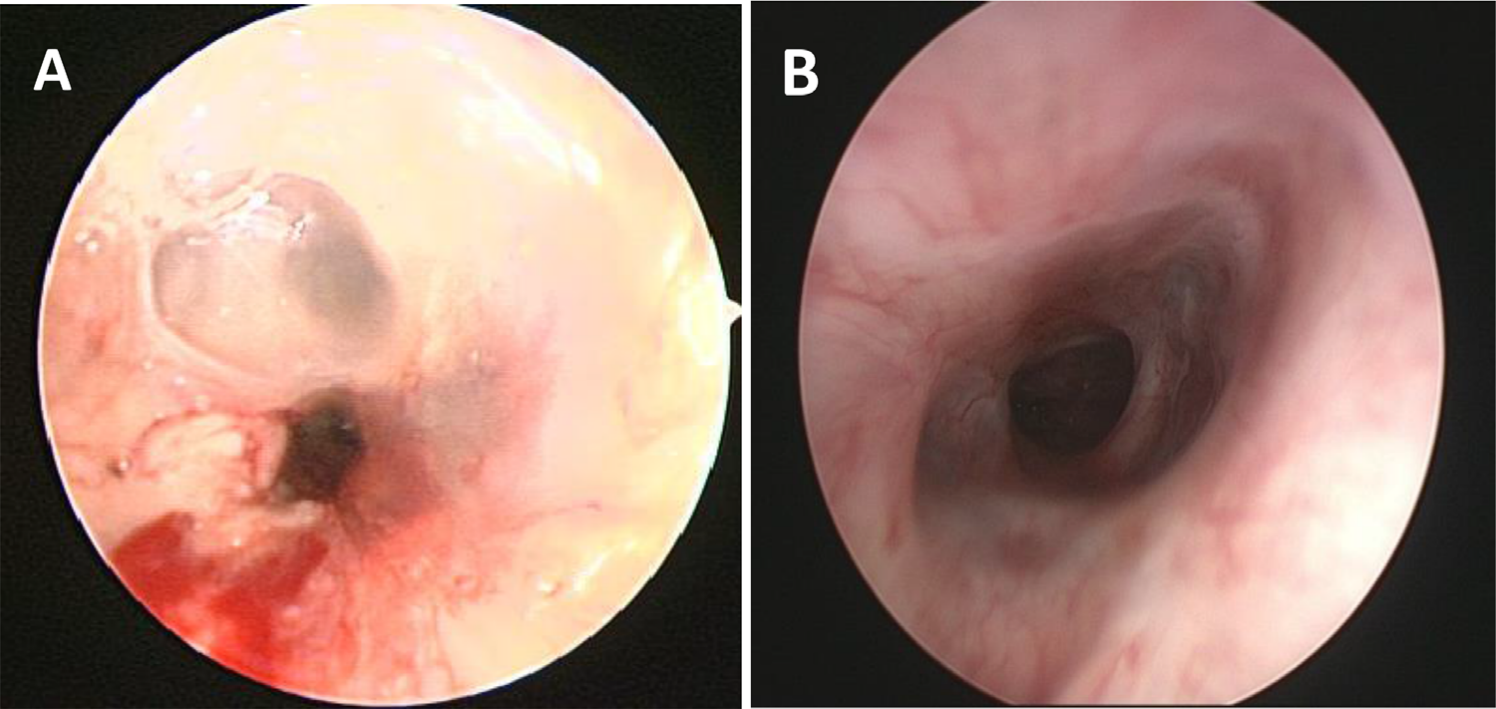
**Bronchoscopy appearances.** (A) Microlaryngoscopy 15 days after transplantation demonstrates a dense web and inflammation within the transplanted segment partially occluding the airway. (B) At 42 months after surgery a complete mucosal layer can be seen within the transplanted segment and a widely patent airway is seen.

**Figure 5:**
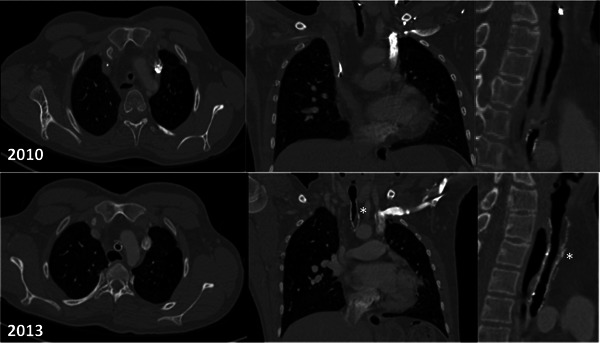
**Computer tomography images: axial, coronal, and sagittal sections taken in 2010 and 2013.** The stents can be seen embedded within the tracheal wall in the 2013 scans (*). The narrowed transplanted segment is visible in both the 2010 and 2013 images. The left sided superior vena cava is not unusual in patients with long segment congenital tracheal stenosis.

Microlaryngoscopy 15 days after transplantation showed a dense web covering the stent and partially occluding the lumen (Figure 4A). At 42 months, a complete mucosal layer is demonstrated throughout the trachea (Figure 4B) with the stents embedded beneath (Figure 5).

**Figure 6:**
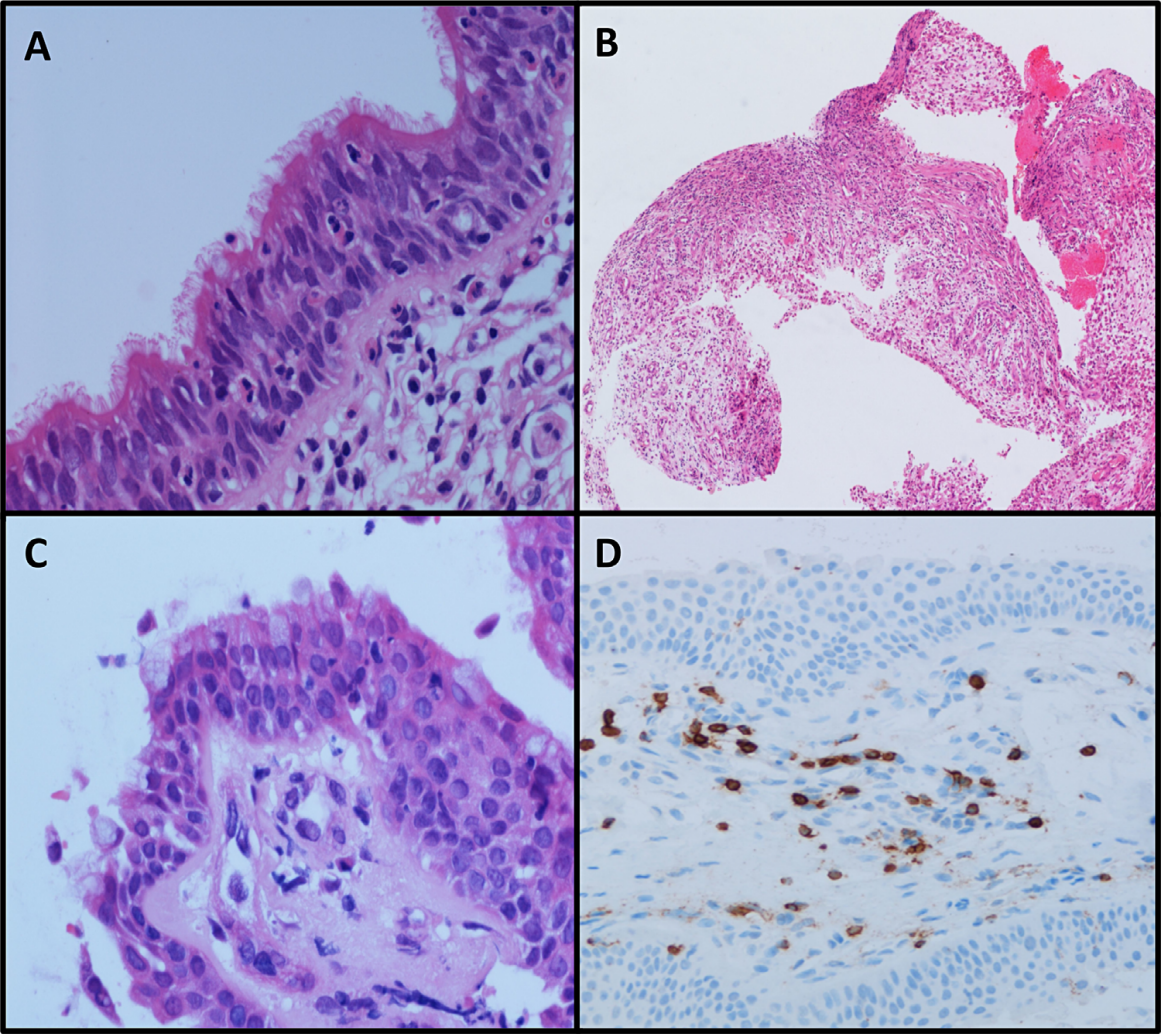
Photomicrographs demonstrating (A) resected homograft trachea March 2010, (B) biopsy of tissue engineered tracheal lining 1 month after transplantation showing extensive granulation tissue, (C) biopsy of proximal tissue‐engineered trachea at 42 months showing complete re‐epithelialization and with scanty ciliated cells, (D) CD3 immunostain for T‐lymphocytes on tracheal biopsy (42 months) demonstrating normal submucosal T cell density.

A section of the excised homograft trachea exhibited normal ciliated respiratory type epithelium (Figure 6A). A biopsy of the transplanted trachea 1‐month following the procedure showed granulation tissue only (Figure 6B). At 42 months, a biopsy of the proximal transplant showed a complete epithelial layer with a mix of squamoid and respiratory type epithelium with scanty ciliated cells (Figure 6C). An immunostained section using CD3 demonstrated no evidence of rejection or lymphocyte‐associated epithelial damage and normal submucosal T cell density (Figure 6D). Staining for Ki67 was normal and cleaved caspase 3 was negative in both pre and posttransplant specimens. Serial serological examination to date has shown no evidence of anti‐donor HLA antibodies.

Brushings on routine endoscopy in April 2014 demonstrated ciliated epithelial cells. Ciliary beat frequency was within the normal range at 10.7 Hz (95% confidence intervals 10 · 4–11 · 0) with a normal ciliary beat pattern. On electron microscopy, structurally normal ciliary axonemes were observed.

### Cost of treatment

**Figure 7:**
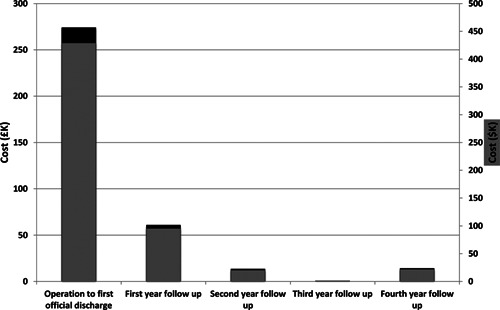
Chart showing the cost of clinical treatment within the initial postoperative period to the fourth year of follow‐up. Clinical costs fell significantly after the initial admission and further still after the first year. Costs slightly increased in the fourth year due to an illness requiring dilatation of an old left bronchial stent.

The total clinical cost of the transplant and follow‐up care to March 2014 was calculated to be US$565 414 (£/$ exchange rate 1:1.56) (Table 1). The costs for treatment from the date of transplant up to first discharge from GOSH contributed to 76% of the total clinical cost, at US$427 255. Following discharge, the clinical costs were less at US$94 749, US$20 693, under US$1560 and US$21 835 for the first, second, third and fourth years, respectively (Figure 7). The laboratory costs of preparing the tissue‐engineered trachea were estimated to be US$15 600.

**Table 1:** Interventions and corresponding costings for first period, i.e., first admission to first official discharge

	Cost ($ approximately)
Ward and ICU stays	$157 265
Imaging	$333
Broncoscopies	$1939
Surgery costs^1^	$268 881
Total	$428 418

ICU, intensive care unit; MLB, microlaryngoscopy and bronchoscopy.

^1^ Including MLBs, stent insertion and removal, bone marrow aspiration for cell harvest, and the procedures involved in the graft transplant itself.

## Discussion

The use of a tissue‐engineered tracheal transplant is currently a potential treatment of last resort. In the reported case, all conventional therapies had failed and the technique was used on compassionate grounds in an urgent setting. This intervention has not only preserved life for more than 4 years, but has enabled the child to mature, continue education and be free of medical intervention for long periods. Indeed, the timing and frequency of interventions between the 1st and 4th years following transplantation compare favorably to children suffering from recurrent tracheal stenosis or those with metallic tracheal stents treated in our institution.

The Peds QL score is consistent with a child suffering from a complex chronic disease 13. A low physical subscale mainly accounted for this and his co‐existing conditions, including spastic diplegia, make it difficult to determine the contribution of his airway disease alone to this score.

The emergent nature of our case necessitated the use of intraoperative native airway epithelial patches to re‐epithelialize the graft 4. The absence of a safe and effective method of monitoring epithelial fate *in vivo* in humans prohibits observations of the fate of such epithelium. A number of preclinical studies have indicated re‐epithelialization occurs from migration of cells from the wound edge following tracheal transplantation 14, 15, tracheal replacement with aortic grafts 16, 17 or synthetic material 18. While this might question the need for re‐epithelizing grafts prior to transplantation, evidence exists supporting the role of epithelium in reducing postoperative stenosis and it is probable that transplanted epithelia act as a biological dressing as re‐epithelization occurs from the wound edge 19.

Alternative protocols for epithelizing tracheal grafts use cadaveric tracheal cartilage prevascularized within radial forearm fascia and lined with autologous buccal mucosa 20, 21. While this approach has reported some success, it is limited by the need for prolonged periods of immunosuppression and the delivery of squamous rather than ciliated epithelium within the airway. The emergent circumstances of our case meant this approach was unsuitable as there was insufficient time for prevascularisation. It is hoped with advances in cell expansion techniques and biomaterials that it will be possible to deliver a fully differentiated autologous respiratory epithelial sheet as has been reported with buccal and corneal epithelium 22, 23. This, in combination with a prevascularised decellularized tracheal scaffold, would allow for the delivery of a vascularized epithelized tracheal graft without immunosuppression.

A cross‐sectional centerline analysis suggested growth at the proximal and distal portions of the trachea over time. Cross‐sectional area is restricted at 15–45 mm below the first tracheal ring in all scans corresponding to an area of in‐stent stenosis. It is not possible to determine whether the transplanted trachea would have grown had in‐stent stenosis been avoided. Whether the transplanted section includes viable tracheal cartilage can also not be determined with routine investigations. However, a complete mucosal layer, dynamic movement of the airway and demonstrable tracheal rings on endoscopy indicate the transplanted trachea is functioning as a cartilaginous frame. Of note, recurrent stenosis has been reported in the other example of a decellularized airway transplant 1.

We also report, for the first time to our knowledge, the use of computational fluid dynamic simulations within a reconstructed trachea. This validated technique provides detailed information on how distinct geometrical features correlate with airflow distribution and provides information on pressure 7, 8, 12. Our results demonstrate an increasing pressure drop at the distal segment of the transplanted trachea due to the jet shown in Figure 3 and highlighted the area most in need of intervention with a corresponding improvement in symptomatology following targeted treatment. We hypothesize that similar results can be derived from high‐field‐strength magnetic resonance imaging using these techniques to provide accurate, personalized and noninvasive planning, and follow‐up following large airway surgery, including transplantation.

The total cost of this tissue‐engineered tracheal transplant is greater than conventional forms of airway management; however, our approach is only indicated for a small subset of children with airway stenosis and the calculated costs herein are unlikely to be prohibitive within a modern healthcare system. Our observations and interpretations of the problems the child faced in this early period have fed back into substantially improved tissue‐engineering protocols, and an advanced GMP process which will lead to substantial, iterative, reductions in hospital stay, complications and, thus, costs.

To our knowledge, this is the first long‐term follow‐up report of a child receiving a tissue‐engineered trachea. Further research is required to develop bioengineered pediatric tracheal replacements with lower morbidity, better biomechanics and at a cost acceptable to healthcare providers. Future clinical trials of such constructs should include comprehensive assessments of airway physiology, biology, quality of life, and health economics.

## Disclosure

The authors of this manuscript have no conflicts of interest to disclose as described by the *American Journal of Transplantation*.

## Supporting information


**Supplementary Materials and Methods**

## References

[1] Gonfiotti A , Jaus MO , Barale D , et al. The first tissue‐engineered airway transplantation: 5‐year follow‐up results. Lancet 2014; 383: 238–244. 24161821 10.1016/S0140-6736(13)62033-4

[2] Macchiarini P , Jungebluth P , Go T , et al. Clinical transplantation of a tissue‐engineered airway. Lancet 2008; 372: 2023–2030. 19022496 10.1016/S0140-6736(08)61598-6

[3] Jungebluth P , Alici E , Baiguera S , et al. Tracheobronchial transplantation with a stem‐cell‐seeded bioartificial nanocomposite: A proof‐of‐concept study. Lancet 2011; 378: 1997–2004. 22119609 10.1016/S0140-6736(11)61715-7

[4] Elliott MJ , De Coppi P , Speggiorin S , et al. Stem‐cell‐based, tissue engineered tracheal replacement in a child: A 2‐year follow‐up study. Lancet 2012; 380: 994–1000. 22841419 10.1016/S0140-6736(12)60737-5 PMC4487824

[5] Griscom NT , Wohl ME . Dimensions of the growing trachea related to age and gender. AJR Am J Roentgenol 1986; 146: 233–237. 3484568 10.2214/ajr.146.2.233

[6] Varni JW , Seid M , Rode CA . The PedsQL: Measurement model for the pediatric quality of life inventory. Med Care 1999; 37: 126–139. 10024117 10.1097/00005650-199902000-00003

[7] Piccinelli M , Veneziani A , Steinman DA , Remuzzi A , Antiga L . A framework for geometric analysis of vascular structures: Application to cerebral aneurysms. Ieee T Med Imaging 2009; 28: 1141–1155. 10.1109/TMI.2009.2021652 19447701

[8] De Backer JW , Vos WG , Vinchurkar SC , et al. Validation of computational fluid dynamics in CT‐based airway models with SPECT/CT. Radiology 2010; 257: 854–862. 21084417 10.1148/radiol.10100322

[9] Yin Y , Choi J , Hoffman EA , Tawhai MH , Lin CL . A multiscale MDCT image‐based breathing lung model with time‐varying regional ventilation. J Comput Phys 2013; 244: 168–192. 23794749 10.1016/j.jcp.2012.12.007 PMC3685439

[10] Chilvers MA , Rutman A , O'Callaghan C . Functional analysis of cilia and ciliated epithelial ultrastructure in healthy children and young adults. Thorax 2003; 58: 333–338. 12668798 10.1136/thorax.58.4.333 PMC1746630

[11] ICS Levels of Critical Care for Adult Patients. No date [cited 2014 Aug 13]. Available from: https://www.rcn.org.uk/_data/assets/pdf_file/0005/435587/ICS_Levels_of_Critical_Care_for_Adult_Patients_2009.pdf

[12] Choi J , Tawhai MH , Hoffman EA , Lin CL . On intra‐ and intersubject variabilities of airflow in the human lungs. Phys Fluids 2009; 21: 101901. 10.1063/1.3247170 PMC2774343 19901999

[13] Varni JW , Seid M , Kurtin PS . PedsQL 4.0: Reliability and validity of the Pediatric Quality of Life Inventory version 4.0 generic core scales in healthy and patient populations. Med Care 2001; 39: 800–812. 11468499 10.1097/00005650-200108000-00006

[14] Genden EM , Iskander A , Bromberg JS , Mayer L . The kinetics and pattern of tracheal allograft re‐epithelialization. Am J Respir Cell Mol Biol 2003; 28: 673–681. 12760965 10.1165/rcmb.2002-0214OC

[15] Ito Y , Suzuki H , Hattori Y , et al. Complete replacement of tracheal epithelia by the host promotes spontaneous acceptance of orthotopic tracheal allografts in rats. Transplant Proc 2004; 36: 2406–2412. 15561264 10.1016/j.transproceed.2004.06.028

[16] Martinod E , Seguin A , Holder‐Espinasse M , et al. Tracheal regeneration following tracheal replacement with an allogenic aorta. Ann Thorac Surg 2005; 79: 942–948; discussion 9. 15734409 10.1016/j.athoracsur.2004.08.035

[17] Makris D , Holder‐Espinasse M , Wurtz A , et al. Tracheal replacement with cryopreserved allogenic aorta. Chest 2010; 137: 60–67. 19801581 10.1378/chest.09-1275

[18] Schultz P , Vautier D , Egles C , Debry C . Experimental study of a porous rat tracheal prosthesis made of T40: Long‐term survival analysis. Eur Arch Rhinolaryngol 2004; 261: 484–488. 10.1007/s00405-003-0717-5 14655018

[19] Go T , Jungebluth P , Baiguero S , et al. Both epithelial cells and mesenchymal stem cell‐derived chondrocytes contribute to the survival of tissue‐engineered airway transplants in pigs. J Thorac Cardiovasc Surg 2010; 139: 437–443. 19995663 10.1016/j.jtcvs.2009.10.002

[20] Delaere P , Vranckx J , Verleden G , De Leyn P , Van Raemdonck D , Leuven Tracheal Transplant G . Tracheal allotransplantation after withdrawal of immunosuppressive therapy. N Engl J Med 2010; 362: 138–145. 20071703 10.1056/NEJMoa0810653

[21] Delaere PR . Tracheal transplantation. Currt Opin Pulm Med 2012; 18: 313–320. 10.1097/MCP.0b013e3283539673 22498734

[22] Moharamzadeh K , Colley H , Murdoch C , et al. Tissue‐engineered oral mucosa. J Dent Res 2012; 91: 642–650. 22266525 10.1177/0022034511435702

[23] Mi S , Connon CJ . The formation of a tissue‐engineered cornea using plastically compressed collagen scaffolds and limbal stem cells. Methods Mol Biol 2013; 1014: 143–155. 23690010 10.1007/978-1-62703-432-6_9

