# Supplementary material for: Tissue‐Engineered Tracheal Replacement in a Child: A 4‐Year Follow‐Up Study
Source: Am J Transplant. 2015 Jun 2;15(10):2750–7. doi: 10.1111/ajt.13318 (PMC4737133; doi:10.1111/ajt.13318)
Supplement: Supplementary file 1 — Supplementary Materials and Methods [file AJT-15-2750-s001.docx]

Supplementary Materials and Methods

3D reconstructions of the tracheal lumen were performed from three CT scans using Mimics^®^ Innovation Suite (Materialise, Materialise Belgium – Technologielaan 15, Leuven, Belgium). A tracheal centreline was generated from the surface of the reconstructed geometry using the Vascular Modelling Toolkit (VMTK, 1·2, http://www.vmtk.org, Copyright © 2013-2014 S.Manini and L.Antiga.) following methods previously described.^7^ An in-house Matlab^®^ R2014a (8·3·0·532), (MathWorks Inc, Natick, MA, US) code generated a plane normal to the local centreline direction at each point. The plane boundary was found where it intersected the surface geometry, allowing cross-sectional area to be calculated. Tracheal airflow was calculated using a validated method through computational fluid dynamics, using Star CCM+^®^ 8·07·004-r8 (CD-adapco, Melville, NY) for both mesh generation and solving the Navier-Stokes equations.^10^ Quasi-steady simulations were performed with a flow rate of 392 ml s^-1^, correlating to the peak flow rate for normal breathing at a tidal volume of 500 ml.^9^
